# The complete mitochondrial genome of *Liparis ochotensis* and a preliminary phylogenetic analysis

**DOI:** 10.1080/23802359.2019.1711217

**Published:** 2020-01-16

**Authors:** Han-Kyeol Sim, Ju-Hyung Jeon, Jeong-Nam Yu, Hyung-Joo Jin, Yong-Ki Hong, Deuk-Hee Jin

**Affiliations:** a Department of Marine Molecular Bioscience, Gangneung-Wonju National University, Gangneung, Korea;; b Biodiversity Conservation and Change Division, Freshwater Biodiversity Research Bureau, Nakdonggang National Institute of Biological Resource (NNIBR), Sangju-Si, Korea;; c Department of Biotechnology, Pukyong National University, Namgu, Korea

**Keywords:** *Liparis ochotensis*, mitochondrial genome, phylogenetic analysis

## Abstract

*Liparis ochotensis* is a snailfish commonly confused with similar fish species because of unclear morphological characteristics. Moreover, molecular genetic studies have not been conducted for snailfish in Korea. Here, we report the complete mitogenome sequence of *L. ochotensis*, obtained via long PCR using universal primers for the fish mitogenome. The *L. ochotensis* mitogenome is 17,522 bp long, comprising 13 protein-coding genes, 22 tRNA genes, two rRNA genes, and one control region. A neighbour-joining phylogenetic tree based on CO1 sequences depicted a close relationship with *Liparis gibbus*. The complete mitogenome is a valuable resource to classify and conserve *L. ochotensis*.

The snailfish, *Liparis ochotensis*, usually caught in Korean East Sea during wintertime, is becoming popular as a unique food. *Liparis ochotensis* tends to be confused with other similar species such as blackmouth owing to similar morphological characteristics. Furthermore, detailed molecular genetic studies, including mitogenome analysis, have not been performed in snailfish. Therefore, we aimed to characterize the mitochondrial genome of *L. ochotensis* from Korea as a first step to elucidate the genetic characteristics of this species and enable accurate taxonomic delineations.

The specimen was collected near Jumunjin Port (N37°53′31.9″, E128°50′57.5″), Gangwon Province, Korea, and stored in the fish collection(GWNU-P-MMB1200007) of the Laboratory of Marine Molecular Biology at Gangneung-Wonju National University, Korea. PCRs using universal primer sets were designed for the fish mitochondrial genome (Miya and Nishida 1999). Biomedic Corp. (Seoul, Korea) performed the analysis using primer walking. The sequences were assembled, aligned, and annotated using MEGA6.0 and tRNAscan-SE 2.0. The sequences of *L. ochotensis* with the annotated genes were deposited in GenBank under the accession number MG718032. The complete mitochondrial genome was similar to that of most other vertebrates (Chen et al. 2012; Li et al. 2012): 17,522 bp long, containing 13 typical vertebrate protein-coding genes (PCGs), 22 tRNA genes, two rRNA genes, and a control region (D-loop).

Except for ND6 and seven tRNA genes, all the other mitochondrial genes are encoded by the heavy strand. This is identical to the typical mitochondrial composition of most fish species (Hurst et al. 1999; Peng et al. 2006; Pie et al. 2017). The base contents constituting 13 CDSs were 57% for A + T and 43% for G + C. Most PCGs started with ATG, except CO1, which started with GTG. Stop codons, including three complete codons (TAA, TAG, AGA) and the incomplete codon T–– of Cytb, were used (Ojala et al. 1981; Boore 1999). Thirteen overlaps were present in the range of about 1–10 bp, including the universal overlap between the four CDSs (ATPase8/ATPase6, ATPase6/CO3, ND4L/ND4, ND5/ND6) (Tzeng et al. 1992; Chang et al., 1994; Zardoya et al. 1995; Boore 1999; Saccone et al. 1999; Hwang et al. 1999). We analyzed the phylogenetic relationships among nine species of Liparidae using CO1 partial gene sequences (633 bp). *Liparis ochotensis* was located at genus *Liparis* with a bootstrap value of up 60% (Figure 1). However, the information currently available suggests a lack of strong support for monophyletic *Liparis* genus. A decisive conclusion cannot be made here, considering that our analysis covered only a small portion of the taxonomic diversity within Liparidae and that a possible sister taxon to the genus *Liparis* is unavailable from the complete mitogenome. Thus, a larger number of complete mitogenome sequences encompassing more taxonomic diversity will be required in future studies. Nevertheless, the present study provides complete mitochondrial genomic data of *L. ochotensis*, which will serve as a valuable resource for phylogenetic and genetic investigations of the Liparidae and taxonomic clarifications.

**Figure 1.**
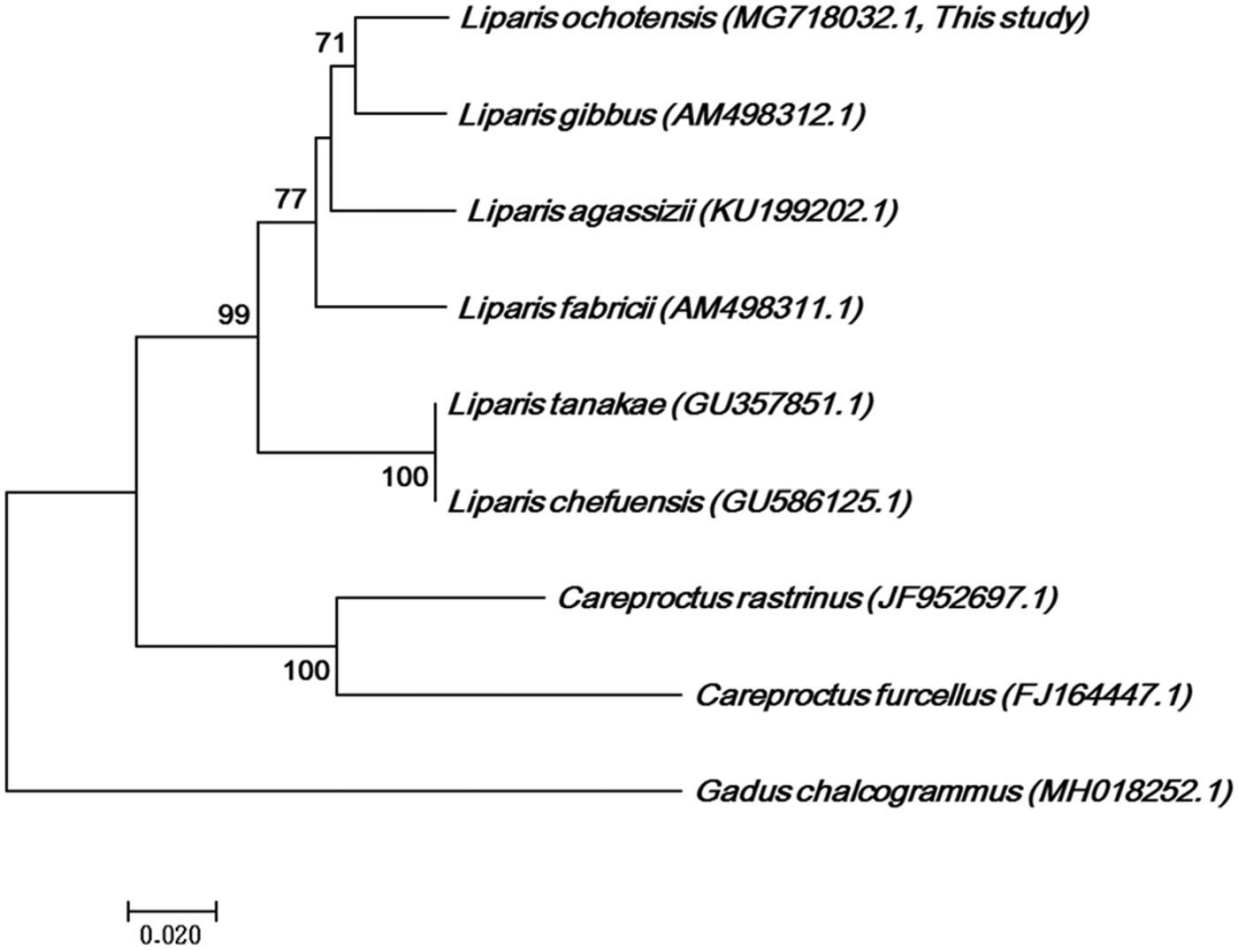
Phylogenetic tree of *Liparis ochotensis* and related species’ complete mtDNA. Neighbour-joining tree based on the CO1 partial gene. The numbers at the nodes are bootstrap values computed using 10,000 replications and Kimura’s 2-parameter distance model. The scale bar indicates 0.02 substitutions per nucleotide position.
